# Endoscopic ultrasound‐guided fine‐needle biopsy for the diagnosis of gastric linitis plastica

**DOI:** 10.1002/deo2.38

**Published:** 2021-09-02

**Authors:** Kosuke Takahashi, Ichiro Yasuda, Tatsuyuki Hanaoka, Yuka Hayashi, Yasuhiro Araki, Iori Motoo, Shinya Kajiura, Takayuki Ando, Haruka Fujinami, Kazuto Tajiri, Masami Minemura, Terumi Takahara

**Affiliations:** ^1^ Third Department of Internal Medicine University of Toyama Toyama Japan

**Keywords:** biopsy, endoscopic ultrasound‐guided fine‐needle biopsy, Franseen‐tip needle, linitis plastica, ultrasonography

## Abstract

We report two cases of patients with gastric linitis plastica (GLP), in which the histopathological diagnosis was made by endoscopic ultrasound‐guided fine‐needle biopsy (EUS‐FNB) using a Franseen‐tip needle. Esophagogastroduodenoscopy findings showed mucosal swelling and poor distensibility of the gastric antrum. Abdominal computed tomography findings showed significant thickening of the gastric wall at the antrum. Conventional endoscopic and bite‐on‐bite biopsy were attempted but resulted in failure to diagnose the lesions. We performed EUS‐FNB to obtain histopathological samples from a deeper site, which confirmed the diagnosis. We considered this method safe and effective for the diagnosis of GLP.

## INTRODUCTION

Gastric linitis plastica (GLP), known as “leather bottle stomach,” is a type of diffuse infiltrating gastric cancer characterized by thickening and rigidity of the gastric wall. In such cases, tumor cells exist more abundantly in the submucosa than in the mucosa. When there are no tumor cells in the mucosa, mucosal ulceration or erosion is often absent.^1^ Patients with other diseases, such as malignant lymphoma, and benign diseases (i.e., Ménétrier's gastritis, lymphoid hyperplasia, and amyloidosis) may present with a thickened gastric wall similar to GLP.^1^ Therefore, it is difficult to distinguish them from GLP based only on endoscopic images. Conventional mucosal biopsy is usually attempted for the diagnosis, however, the result is often false negative because the tumor cells occupy deeper sites. Therefore, sampling of the deeper tissues is necessary for the diagnosis.

In this report, we present two cases of patients with GLP, in which endoscopic ultrasound‐guided fine‐needle biopsy (EUS‐FNB) with a Franseen‐tip needle was used to successfully confirm the diagnosis after a failed conventional endoscopic biopsy. All participants provided written informed consent to undergo the procedure. The Toyama University Hospital Institutional Review Board granted permission to review the patients’ records. The study was conducted according to the principles of the Declaration of Helsinki.

## CASE REPORT

Case 1 was of a 68‐year‐old woman who underwent esophagogastroduodenoscopy (EGD) at a local clinic because of epigastric discomfort. EGD findings showed mucosal swelling and poor distensibility at the gastric antrum. Conventional endoscopic biopsy was performed, resulting in an inconclusive pathological diagnosis. Therefore, the patient was referred to our hospital for further evaluation.

**FIGURE 1:**
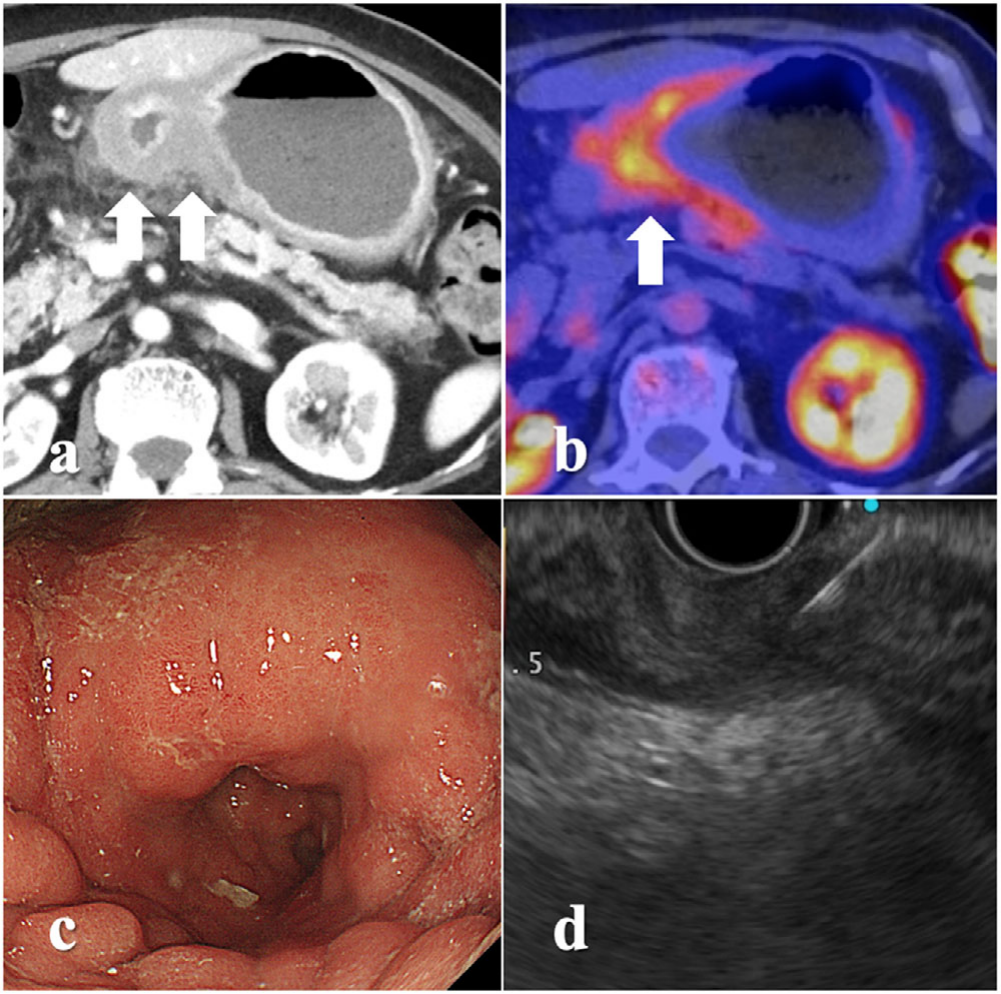
Findings of investigations performed in a 68‐year‐old female patient: (a) CT findings showing thickening of the gastric wall at the antrum (arrow); (b) fluorodeoxyglucose‐positron emission tomography‐computed tomography (FDG‐PET‐CT) showing slight accumulation of FDG (standardized uptake value (SUV); max: 6.36) in the gastric antrum (arrow); (c) esophagogastroduodenoscopy (EGD) showing luminal stricture without any mucosal findings; (d) endoscopic ultrasound‐guided fine‐needle biopsy (EUS‐FNB) was performed to collect biopsy specimen from the thickened gastric wall

**FIGURE 2:**
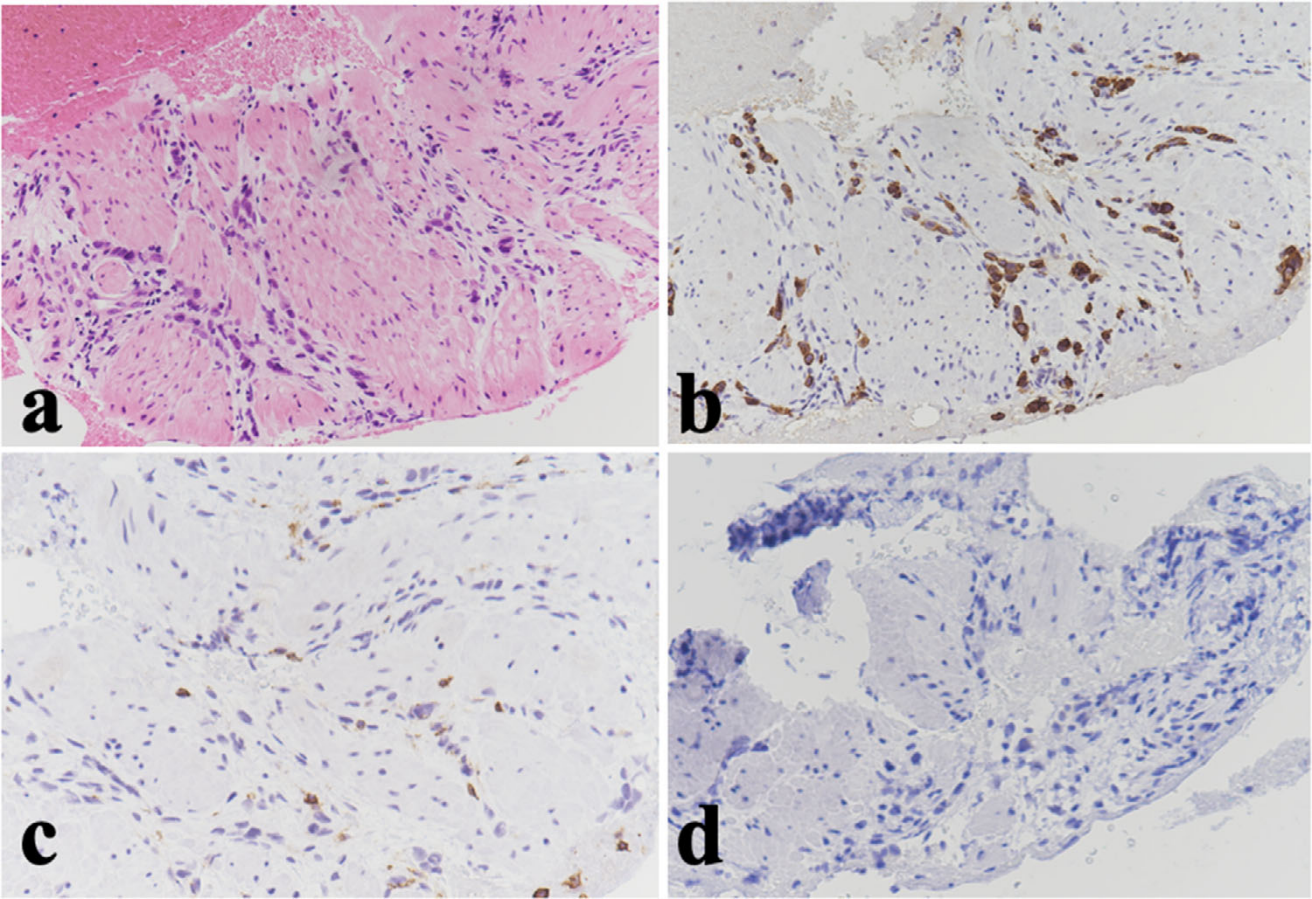
Histopathological findings of an endoscopic ultrasound‐guided fine‐needle biopsy (EUS‐FNB) specimen from a 68‐year‐old female patient: (a) hematoxylin and eosin staining; (b) positive immunohistochemistry (IHC) staining for cytokeratin AE1/AE3; (c) negative IHC staining for leukocyte common antigen (LCA); (d) HER2

The serum carcinoembryonic antigen and cancer antigen (CA) 125 levels were within the normal range, however, the CA 19‐9 level was elevated to 343 U/ml. Abdominal computed tomography (CT) findings showed thickening of the gastric wall at the antrum (Figure 1a). Fluorodeoxyglucose‐positron emission tomography‐CT (FDG‐PET‐CT) findings showed FDG accumulation (SUV [standardized uptake value] max: 6.36) at the gastric antrum (Figure 1b). Moreover, EGD findings showed stricture without mucosal changes (Figure 1c). Although several biopsies using the bite‐on‐bite technique were performed, a pathological diagnosis could not be made. Therefore, we attempted to perform EUS‐FNB for the histopathological diagnosis. The gastric wall was thickened up to 9.3 mm at the gastric antrum on EUS. The gastric wall was punctured using a 22‐gauge Franseen‐tip needle (AcquireTM; Boston Scientific Co., Natick, MA, USA) (two sessions, 10 strokes each using a 10‐cc syringe for negative pressure; Figure 1d). Histopathological findings showed poorly differentiated adenocarcinoma in the muscularis propria layer. Immunohistochemistry findings revealed cytokeratin AE1/AE3‐positive and leukocyte common antigen (LCA) and HER2‐negative cells (Figure 2). The definitive diagnosis of gastric cancer was made based on these histopathological findings.

The patient had Stage‐III cancer as per the Union for International Cancer Control (UICC) classification (T4aN1M0). Despite our recommendation, she was unwilling to undergo surgery; therefore, general chemotherapy was commenced later.

**FIGURE 3:**
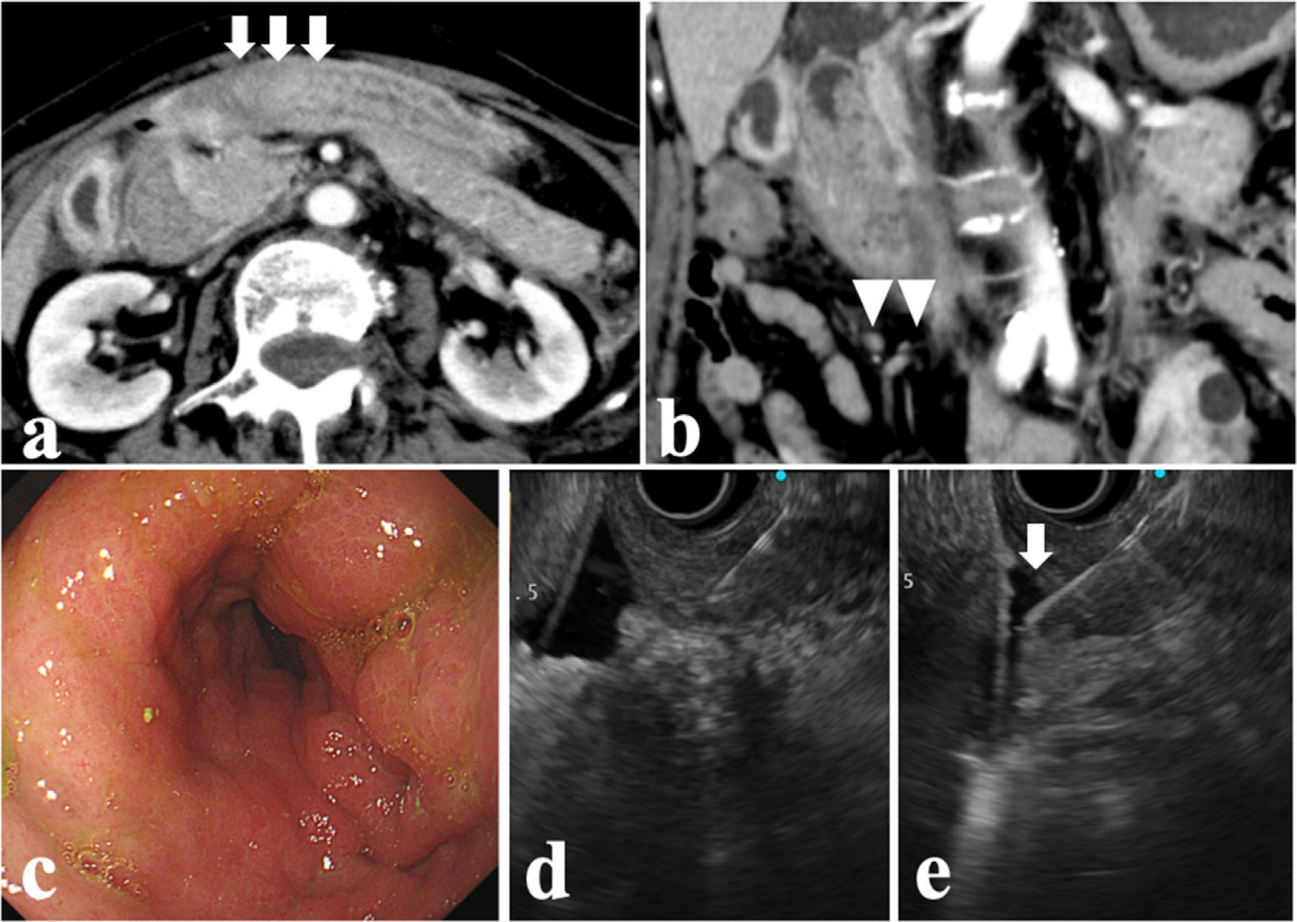
Findings of investigations performed in a 69‐year‐old female patient: (a) computed tomography (CT) findings showing a circumferential thickening of the gastric wall at the antrum (arrow); (b) multiple nodules were also observed in the abdominal cavity (arrowheads); (c) esophagogastroduodenoscopy (EGD) findings showing luminal rigid stricture without mucosal erosion and ulcers; (d) endoscopic ultrasound‐guided fine‐needle biopsy (EUS‐FNB) was performed to obtain a sample from the thickened gastric wall; (e) ascites were also aspirated (arrow)

Case 2 was of a 69‐year‐old woman who visited a local hospital because of anorexia. EGD was performed at the hospital, which showed mucosal swelling and poor distensibility of the gastric antrum. Conventional endoscopic mucosal biopsy was performed with histopathological findings yielding an inconclusive diagnosis. However, CT showed circumferential thickening of the gastric wall at the gastric antrum and multiple nodules in the abdominal cavity (Figure 3a and b). Therefore, she was referred to our hospital for further evaluation.

**FIGURE 4:**
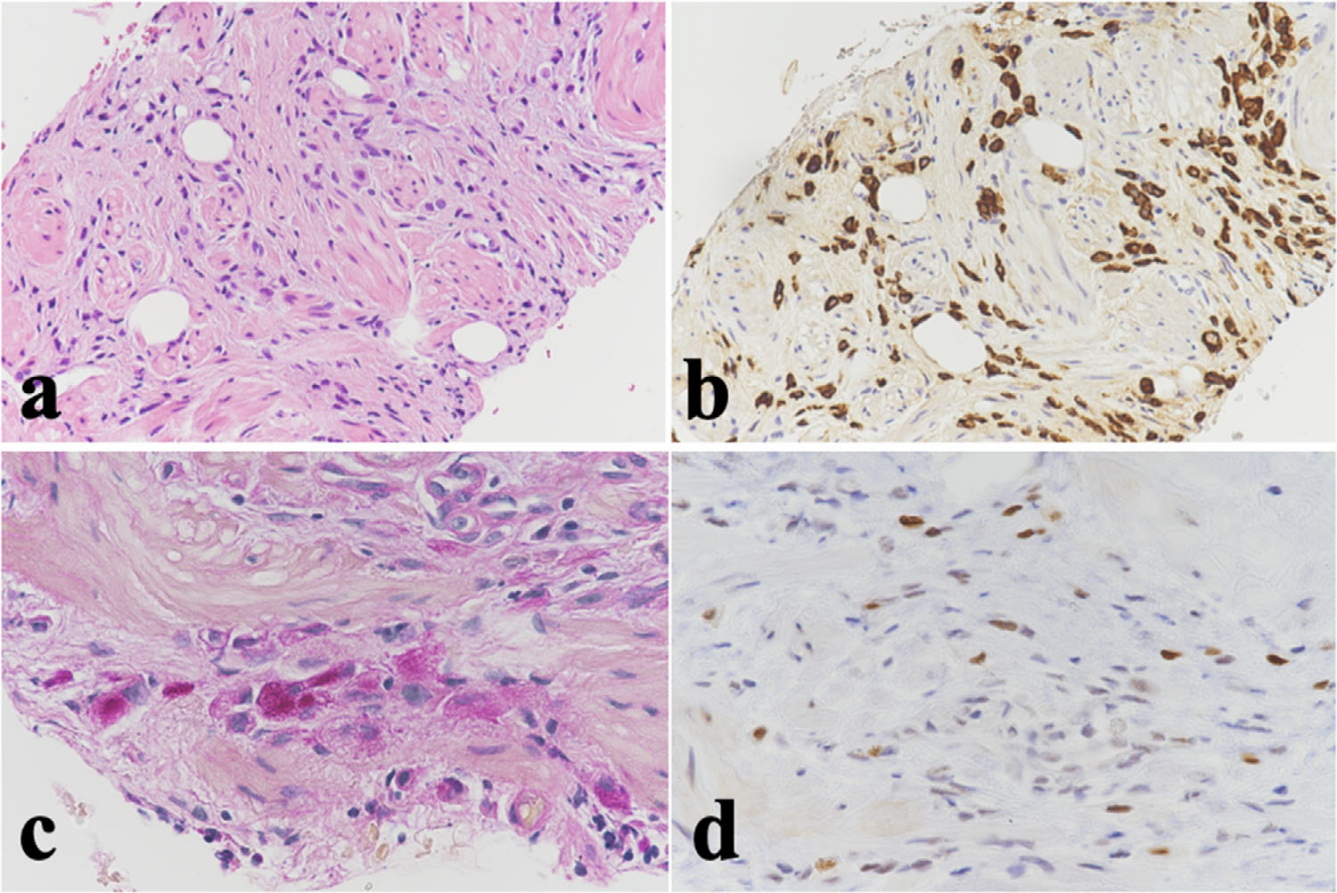
Histopathological findings of an endoscopic ultrasound‐guided fine‐needle biopsy (EUS‐FNB) specimen obtained from a 69‐year‐old female patient: (a) hematoxylin and eosin staining; (b) positive immunohistochemistry (IHC) staining for cytokeratin AE1/AE3; (c) positive periodic acid‐Schiff (PAS) staining; (d) positive IHC staining for p53.

The blood test findings were normal, including the carcinoembryonic antigen and CA 19‐9 levels, except for a slight elevation in the CA 125 level (73 U/mL). EGD findings showed stricture with rigidity without mucosal erosion and ulceration in the gastric antrum (Figure 3c). Therefore, several biopsies with the bite‐on‐bite technique were performed to obtain the pathological sample from deeper sites. However, the pathological diagnosis was still inconclusive. Subsequently, EUS‐FNB was attempted. EUS findings showed thickening of the gastric wall up to 10.0 mm and small amount of ascites. EUS‐FNB was performed for the gastric wall using a 22‐gauge Franseen‐tip needle using the same method as that performed in Case 1. Ascites was also aspirated via the gastric wall (Figure 3d and e). Histopathological examination revealed that the poorly differentiated adenocarcinoma cells were intermittently infiltrating the smooth muscles. The atypical cells were positive for cytokeratin AE1/AE3 and periodic acid‐Schiff according to the immunohistochemical staining. Moreover, they showed a high expression of MIB‐1 and abnormal p53 accumulation (Figure 4). Furthermore, peritoneal dissemination was confirmed by the cytological assessment of ascites.

The final diagnosis was gastric cancer UICC Stage IVB and general chemotherapy was commenced.

## DISCUSSION

The diagnosis of GLP is often difficult because of its pathological features presenting as submucosal proliferation. Endoscopic findings often show only thickening and rigidity of the gastric wall without any suspicious mucosal findings. In addition, definitive diagnosis of GLP is difficult based only on EUS findings. Although pathological diagnosis is required to establish the definitive diagnosis of GLP, conventional endoscopic forceps biopsy is often difficult to obtain adequate sample, as the tumor cells are found mainly in the submucosal or deeper layers with reactive fibrosis.^1^ Kim et al. showed that the false‐negative rate with endoscopic mucosal forceps biopsy was approximately 55.9% for Bormann type IV gastric cancer.^2^

For biopsy from deeper sites, several endoscopic biopsy techniques are currently used, such as the bite‐on‐bite technique, jumbo biopsy, endoscopic mucosal resection, mucosal incision‐assisted biopsy (MIAB), and EUS‐guided fine‐needle aspiration (EUS‐FNA).^3^ However, jumbo biopsy and endoscopic mucosal resection do not significantly increase the volume depth.^4^ Conversely, MIAB has been reported to be a useful method of diagnosis for subepithelial lesions (SELs). In a previous prospective randomized cross‐over study, the diagnostic yield of a MIAB for SEL was similar to that of EUS‐FNA (91.3% versus 70.8%, *p* = 0.0746). However, the diagnostic yield of a MIAB was significantly higher than that of EUS‐FNA in patients with tumors <2 cm in size, lesions in the greater curvature, and non‐gastrointestinal stromal tumor SELs.^5^ In addition, EUS‐FNA can improve the diagnostic accuracy for GLP without increasing the risk of bleeding and perforation.^3^ Ye et al. reported that the diagnostic accuracy of EUS‐FNA for GLP was 87.5%.^6^ In contrast, Liu et al. reported that EUS‐FNA had no significant advantage compared with white‐light endoscopy‐guided biopsy.^7^ Therefore, it is desirable to develop diagnostic tools that exceed EUS‐FNA.

Recently, EUS‐FNB has gained popularity, as it helped us obtain more pathological samples and easily establish a more accurate pathological assessment. Several studies have already confirmed that the diagnostic ability of EUS‐FNB is superior to that of EUS‐FNA.^8^ In Japan, a needle with three novel symmetrical heels called “Franseen‐tip needle” has become available for EUS‐FNB. Mukai reported that, for histological evaluation, a 22‐gauge Franseen‐tip needle provides approximately a five‐times increased median area of tissue sample compared to the 22‐gauge conventional FNA needle.^9^

In our reported cases, EUS‐FNB was performed because mucosal biopsy and biopsy with the bite‐on‐bite technique could not help in making a definitive diagnosis. Although the thickening of the stomach wall was thin (9.3 and 10.0 mm in Cases 1 and 2, respectively), sufficient sample volume was obtained for histopathological and immunohistochemical examination without any complications. In addition, GLP often metastasizes to the lymph nodes and peritoneum. EUS‐FNB is also helpful in determining the TNM staging using the cytological and histological assessment of such lesions. In our second case, a small amount of ascites was also obtained by EUS‐FNB, and the diagnosis of carcinomatous peritonitis was confirmed using the cytological assessment. The 22‐gauge Franseen‐tip needle may be recommended for EUS‐FNB for GLP because of its easy manipulation and high diagnostic ability.

While EUS‐FNB contributes to the diagnosis of GLP, needle tract seeding should be noted. Since the report of the first case by Hirooka et al. in 2003, 18 cases have been reported to date.^10^ Although they are all derived from pancreatic cancer, performing EUS‐FNB via the stomach for ascites in cases of gastric cancer without cancerous peritonitis may also have a risk of needle tract seeding. Therefore, in cases of puncturing ascites, we should puncture as much as possible from a site without gastric wall thickening where gastric cancer is not suspected.

In conclusion, EUS‐FNB using a Franseen‐tip needle is a safe and effective technique for the definitive diagnosis of GLP when it is being considered as a probable cause of gastric wall thickening.

## CONFLICT OF INTEREST

Author I.Y. is an Associate Editor of DEN Open.

## FUNDING INFORMATION

None.
